# Effects of recall time on cause-of-death findings using verbal autopsy: empirical evidence from rural South Africa

**DOI:** 10.1186/s12982-016-0051-1

**Published:** 2016-10-18

**Authors:** Laith Hussain-Alkhateeb, Max Petzold, Mark Collinson, Stephen Tollman, Kathleen Kahn, Peter Byass

**Affiliations:** 1 Health Metrics, Sahlgrenska Academy, University of Gothenburg, Box 414, 405 30 Gothenburg, Sweden; 2 School of Public Health, Faculty of Health Sciences, University of the Witwatersrand, Johannesburg, South Africa; 3 WHO Collaborating Centre for Verbal Autopsy, Umeå Centre for Global Health Research, Epidemiology and Global Health, Department of Public Health and Clinical Medicine, Umeå University, Umeå, Sweden; 4 INDEPTH Network, Accra, Ghana; 5 Medical Research Council/Wits University Rural Public Health and Health Transitions Research Unit (Agincourt), School of Public Health, Faculty of Health Sciences, University of the Witwatersrand, Johannesburg, South Africa

**Keywords:** HDSS, Verbal autopsy, Cause of death, Recall, Time lapse, South Africa

## Abstract

**Background:**

Verbal autopsy (VA) is a widely used technique for assigning causes to non-medically certified deaths using information gathered from a close caregiver. Both operational and cultural factors may cause delays in follow-up of deaths. The resulting time lag—from death to VA interview—can influence ways in which terminal events are remembered, and thus affect cause-of-death assignment. This study investigates the impact of recall period on causes of death determined by VA.

**Methods:**

A total of 10,882 deaths from the Agincourt Health and Demographic Surveillance System (HDSS) with complete VAs, including recall period, were incorporated in this study. To measure seasonal effect, cause specific mortality fractions (CSMFs) were calculated and compared by every cause for VAs undertaken within six months of death and those undertaken from six to 12 months of death. All causes were classified into eight broad categories and entered in a multiple logistic regression to explore outcome by recall period in relation to covariates.

**Results:**

The majority of deaths (83 %) had VAs completed within 12 months. There was a tendency towards longer recall periods for deaths of those under one year or over 65 years of age. Only the acute respiratory, diarrhoeal and other unspecified non-communicable disease groups showed a CSMF ratio significantly different from unity at the 99 % confidence level between the two recall periods. Only neonatal deaths showed significantly different OR for recall exceeding 12 months (OR 1.69; p value = 0.004) and this increased when adjusting for background factors (OR 2.58; p value = 0.000).

**Conclusion:**

A recall period of up to one year between death and VA interview did not have any consequential effects on the cause-of-death patterns derived, with the exception of neonatal causes. This is an important operational consideration given the planned widespread use of the VA approach in civil registration, HDSS sites and occasional surveys.

## Background

Accurate and timely data on mortality by demographic factors, both nationally and sub-nationally, are essential for developing, monitoring, and evaluating health policies and programmes [1]. Health and Demographic Surveillance Systems (HDSSs) can be effective responses to the lack of systematic registration of vital statistics, particularly in resource-limited settings [2–4]. HDSSs can provide necessary evidence through routine update rounds for monitored populations, including documenting causes of death reliably using verbal autopsy (VA) [5]. Surveys of recent deaths can also be undertaken in unregistered populations using VA [6]. In both approaches, an important question arises as to the time that can reasonably elapse between a death occurring and a VA being undertaken.

To undertake VAs, fieldworkers visit households during HDSS update rounds or in the context of other surveys, usually interviewing the person most knowledgeable about the illness or events preceding a death [7]. In HDSSs, updating and verifying existing data or recording new events such as pregnancy outcomes, in- and out-migrations and other socio-economic features as well as birth and death events are carried out via regular update rounds. These rounds may be annual, or more frequent. VA is a widely used technique for assigning cause to non-certified deaths. It elicits most probable cause of death using a standardised interview instrument following detection of a death, with the data usually then processed automatically to assign likely causes of death [5, 8–10].

There are operational and cultural factors which may cause delays in following-up deaths, and these can be associated with the nature of the death. Longer recall times may influence ways in which original events are remembered, and thus affect responses to specific questions in a VA interview, hence influencing conclusions on causes of death [11, 12].

Since evidence on the effect of recall and its consequences on VA processes is limited [5], this study aims to investigate the effect of recall period on causes of death determined by VA, in order to make recommendations about realistic recall periods for undertaking VAs.

## Methods

### Study setting and data collection

Empirical data on VA recall was sourced from the Agincourt HDSS in rural norteast South Africa, which is described in detail elsewhere [7], and was a founder member of the INDEPTH Network [13]. It continuously surveys around 90,000 inhabitants living in approximately 16,000 households across 31 villages. From 1992 to 2011, routine visits by trained fieldworkers successfully completed 11,187 VAs with next of kin or other caregivers. Standardised interviews in the local language (Shangaan) were used to elicit signs, symptoms and circumstances of the terminal illness as well as individual health-related behaviour. The collected VA data were transformed into the WHO 2012 VA standard [14] and processed using the automated InterVA-4 probabilistic model (version 4.02) [15] which assigns up to three likely causes for each death and is the most widely-used VA tool. A summary of mortality in the Agincourt population from the same dataset is presented in a recent report [16].

The Agincourt HDSS has undertaken annual update rounds since its inception in 1992. Following a baseline census the same year, the annual census round and the VA interviews have been undertaken strictly between August and November (although some VAs continue into December to finish off) which causes the dominant short-period of five months during the dry season. Deaths identified during the update rounds are followed up by specially trained field workers who conduct a VA interview with the closest available caregiver. As a consequence of Agincourt’s annual cycle of operation, there is a usual range from about four weeks (recognised mourning period during which no interview is conducted) to around one year between death and the VA interview, but this can extend to a longer period in a minority of cases, for example if deaths are missed in update rounds.

### Measurement procedures

For the 12,209 deaths in the Agincourt HDSS between 1992 and 2011, a total of 11,187 (91.6 %) had valid VAs completed, as previously reported [17]. The Agincourt HDSS database also has archived information on the deceased’s background and characteristics, but only 10,882/11,187 (97.3 %) had records on the period between death and VA interview, which thus defined the overall dataset for these analyses. Update rounds at the Agincourt follow periodic routine and enhanced by on-site supervision and quality control which occur at five levels; individual-checks (daily), cross-checks (weekly) and random-checks that covers all survey tools and collected data. This is regularly followed by quality checkers and data validation checks to evade and correct associated errors [18].

Relevant individual background characteristics were utilised to adjust for cultural factors that may influence reporting deaths in these communities. The context of death and individuals’ characteristics such as age, education and race; which defines the cultural interpretations of symptomatology of particular illnesses have been reported to influence recall accuracy [10]. Such factors are important to consider and account for in the analysis. Age at death was categorised into five groups [0–11 months (infant), 1–14 years (child), 15–49 years (reproductive age), 50–64 years (adult) and 65+ years (elderly)]. At the time of the Mozambican civil war there was a substantial refugee influx of ethnic Shangaan people from Mozambique into the Agincourt area; they and their direct descendents in Agincourt are referred to as former Mozambican refugees, irrespective of formal nationality. Three levels of education were defined by numbers of years of study the VA respondent has completed (none, 1–7 and 8–15 years). The year of death (1992–2011) and place of death (home, hospital/health facility or other) were also recorded.

Although true cause of death is assumed independent of any time lag, the annual cycle of documenting and following up deaths in the Agincourt population would probably lead to confounding between time of year and recall period, which could be important for causes of death subject to seasonal variation. A variable for season was created which put deaths in the drier months (May to September inclusive) into one category, compared with the wetter months (October to April inclusive), based on meteorological data.

Output from the InterVA-4 model consists of up to three likelihoods per case attributed to different causes, and for individuals where these do not total 100 %, an indeterminate residual, as previously described [17]. Deaths due to any specific cause or cause group can then be determined by summing likelihoods; regression models can use the individual likelihoods as weights in models with multiple records per case.

To investigate the seasonal effect, cause specific mortality fractions (CSMF) for every cause of death in the WHO 2012 VA cause list were calculated separately for VAs undertaken within six months of death and those undertaken from six to 12 months of death. All maternal causes were aggregated into a single category because of small numbers. CSMF ratios for the two time periods and their 99 % confidence intervals were calculated for each cause using the Katz adjusted log method in view of the small numbers involved for some causes [19].

To explore the VA assessment of causes of death by recall period in relation to covariates, all causes were classified into eight broader categories for analysis. Both HIV and pulmonary tuberculosis (TB) commonly co-exist in this population with high degree of symptoms-overlap, especially in younger adults, justifying their combination into one category [20]. Acute respiratory infections were retained as a single category, while all remaining infectious causes, neoplasms, other NCDs (including the few maternal deaths), neonatal deaths and external causes were defined as separate categories. Cases with contradictory or insufficient information for the InterVA-4 model, as well as the residual fractions, were classified as ‘indeterminate’, forming the eighth category. The time between death and VA was stratified into four groups to define the recall period: less than three months, three to five months, six to eleven months and over one year. Logistic regression modelling was used to calculate adjusted odds ratios associated with different recall periods for the eight cause categories, using cause likelihood for each fractional death as a weight, individual ID number to identify all records belonging to an individual, and appropriate covariates as independent factors.

## Results

A total of 10,882 deaths with a completed VA, including data on the period between death and VA interview, were processed. The median recall was 7 months, ranging from the first month after death to 48 months.

**Fig. 1:**
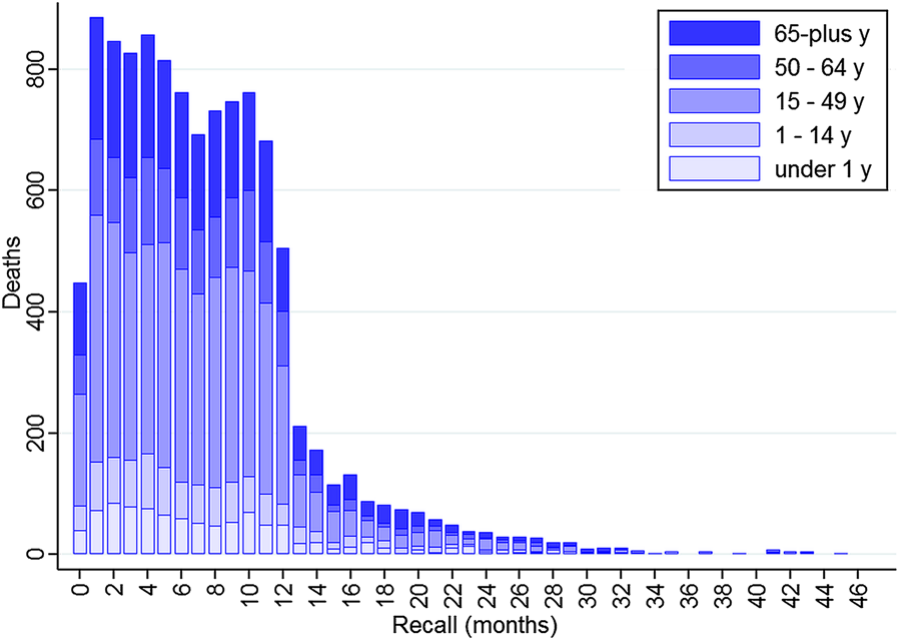
Distribution of deaths by VA recall months and age groups, 1992–2011, Agincourt, South Africa

Figure 1 summarises the distribution of all deaths by the recall month (number of completed months from death to VA), showing that the majority of deaths (83 %) had VAs done within 12 months. This reflects the practice in the Agincourt HDSS of undertaking most VAs during the course of an annual update round and hence no more than 12 months since a death.

**Fig. 2:**
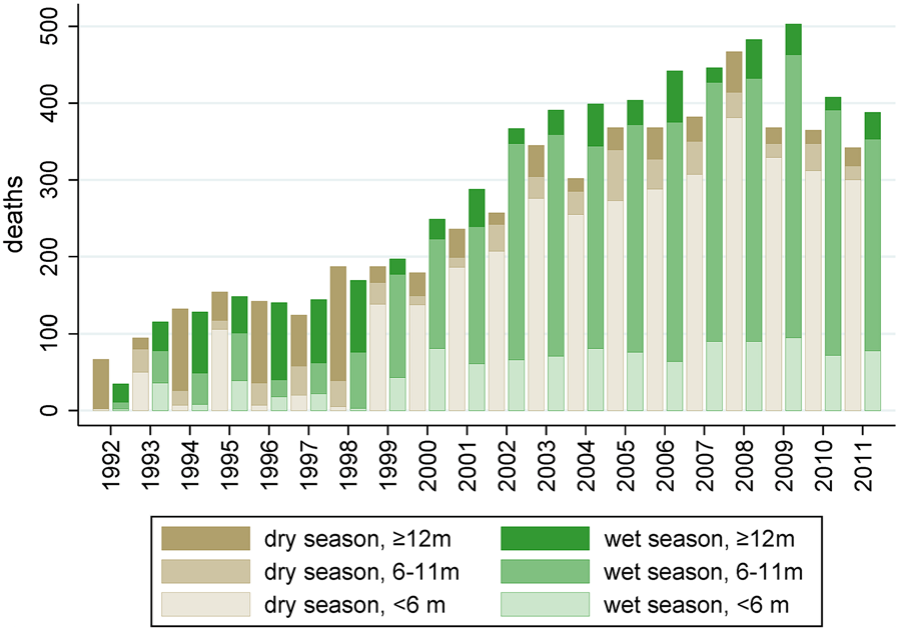
Distribution of mortality by year of death and season (dry/wet), by recall months for VA interviews, 1992–2011, Agincourt, South Africa

Following its establishment in 1992, mortality in the Agincourt HDSS increased steadily as the South African HIV/AIDS epidemic took hold, with decreases only in recent years as anti-retroviral therapy became available, as shown in Fig. 2.

Both figures shed light on strategically important operational aspects during these routine annual rounds of VAs in Agincourt. As evident in the first figure, VAs were less frequently undertaken in the first month after death, giving respect to families in the immediate aftermath of a death. The corollary of this is that some deaths occurring around the time of the annual update round are followed up just over a year later. The age distribution of recall also shown in Fig. 1 showed a tendency towards longer recall periods for deaths of those who were under one year or over 65 years. Figure 2 also shows that recall period was significantly confounded with season, with the majority of recalls under six months relating to deaths in the dry season.

Cause specific mortality fraction ratios for recall periods of less than six months versus 6–11 months are shown in Table 1. The acute respiratory, diarrhoeal and other unspecified NCD groups showed a CSMF ratio significantly different from unity at the 99 % CI level between the two recall periods.

**Table 1:** CSMF ratio by recall periods (0–5 month vs. 6–11 months) with 99 % CI, according to WHO 2012 VA cause of death groups

Cause of death	CSMF ratio	99 % CI
01.01 Sepsis (non-obstetric)	1.61	0.63–4.11
01.02 Acute resp infect. incl pneumonia	1.34	1.13–1.57*
01.03 HIV/AIDS related death	1.03	0.93–1.13
01.04 Diarrhoeal diseases	1.52	1.08–2.14*
01.05 Malaria	0.74	0.48–1.15
01.06 Measles	1.04	0.19–5.67
01.07 Meningitis and encephalitis	1.52	0.71–3.25
01.09 Pulmonary tuberculosis	1.07	0.97–1.19
01.10 Pertussis	0.89	0.36–2.24
01.11 Haemorrhagic fever	1.43	0.14–14.21
01.99 Other and unspecified infect dis	1.13	0.51–2.55
02.01 Oral neoplasms	1.45	0.41–5.07
02.02 Digestive neoplasms	0.83	0.63–1.09
02.03 Respiratory neoplasms	0.87	0.64–1.18
02.04 Breast neoplasms	0.61	0.25–1.49
02.05. 02.06 Reproductive neoplasms M.F	1.29	0.73–2.27
02.99 Other and unspecified neoplasms	1.03	0.70–1.51
03.01 Severe anaemia	1.01	0.07–13.91
03.02 Severe malnutrition	0.80	0.42–1.55
03.03 Diabetes mellitus	1.16	0.86–1.55
04.01 Acute cardiac disease	1.25	0.62–2.53
04.02 Stroke	1.10	0.85–1.43
04.99 Other and unspecified cardiac dis	1.09	0.84–1.42
05.01 Chronic obstructive pulmonary dis	1.12	0.83–1.51
05.02 Asthma	1.23	0.89–1.71
06.01 Acute abdomen	0.91	0.59–1.39
06.02 Liver cirrhosis	1.02	0.50–2.06
07.01 Renal failure	0.72	0.27–1.92
08.01 Epilepsy	1.33	0.60–2.96
09.00 Maternal	0.91	0.42–1.96
10.01 Prematurity	1.12	0.41–3.09
10.02 Birth asphyxia	1.74	0.83–3.65
10.03 Neonatal pneumonia	1.55	0.93–2.61
10.04 Neonatal sepsis	0.85	0.23–3.06
10.06 Congenital malformation	1.40	0.33–5.88
10.99 Other and unspecified neonatal CoD	1.20	0.45–3.21
12.01 Road traffic accident	0.95	0.71–1.29
12.04 Accidental drowning and submersion	0.47	0.14–1.56
12.05 Accidental exposure to smoke, fire & flame	1.31	0.56–3.08
12.07 Accidental poisoning and noxious subs	0.47	0.09–2.37
12.08 Intentional self-harm	0.90	0.55–1.47
12.09 Assault	0.87	0.65–1.16
12.99 Other and unspecified external CoD	0.46	0.14–1.46
98 Other and unspecified NCD	1.75	1.02–2.98*
99 Indeterminate	1.05	0.91–1.20

* CSMF ratio between the 0–5 and 6–11 months recall groups significantly different from unity

The logistic regression analysis assessed patterns of mortality by the broader categories according to recall period. Table 2 presents the crude and adjusted odds ratios (OR) of all cause categories across the recall periods, with the shortest recall period (<3 months) as the reference group. A consistent trend was seen across all the infectious categories, which accounted for 45 % of all deaths. The crude OR for each category was significantly different for recall periods over one year, but these differences did not persist when adjusted for background factors. Neither the neoplasm nor other NCD categories showed significant differences in crude or adjusted ORs. The odds of neonatal deaths were significantly higher with recall exceeding 12 months (OR 1.69; p value = 0.004) compared to the 3 months period and this association increased when adjusting for background factors (OR 2.58; p value = 0.000), for reasons that are not entirely clear. Both external causes and the indeterminate category mirrored the trend shown across the infectious categories; adjusting for background factors reduced differences by recall period.

**Table 2:** Logistic regression analysis to examine the effect of recall period on the VA assessment of all cause of death groups in Agincourt, South Africa between 1992 and 2011

Cause of death	Recall period^a^		
	3–5 months OR (p value)	6–11 months OR (p value)	≥12 months OR (p value)
HIV/TB (*n* = 3759)			
Crude	0.96 (0.423)	1.00 (0.897)	0.66 (0.000)*
Adjusted^b^	0.94 (0.339)	1.01 (0.885)	0.87 (0.065)
Acute respiratory (*n* = 712)			
Crude	0.90 (0.287)	0.74 (0.001)*	0.78 (0.018)*
Adjusted^b^	0.93 (0.467)	0.92 (0.468)	0.88 (0.340)
Other Infectious (*n* = 405)			
Crude	1.10 (0.453)	0.93 (0.564)	1.40 (0.009)*
Adjusted^b^	1.07 (0.605)	0.87 (0.336)	1.03 (0.862)
Neoplasm (*n* = 609)			
Crude	0.90 (0.313)	1.13 (0.165)	1.15 (0.173)
Adjusted^b^	0.85 (0.140)	0.88 (0.247)	0.99 (0.961)
Other NCDs (*n* = 1429)			
Crude	1.10 (0.166)	1.00 (0.990)	0.91 (0.225)
Adjusted^b^	1.10 (0.181)	1.04 (0.584)	1.07 (0.466)
Neonatal (*n* = 187)			
Crude	0.97 (0.876)	0.74 (0.102)	1.69 (0.004)*
Adjusted^b^	1.17 (0.504)	1.49 (0.114)	2.58 (0.000)*
External (*n* = 641)			
Crude	0.95 (0.712)	1.20 (0.074)	1.68 (0.000)*
Adjusted^b^	0.99 (0.936)	1.14 (0.326)	1.27 (0.097)
Indeterminate (*n* = 3140)			
Crude	1.12 (0.085)	1.09 (0.119)	1.56 (0.000)*
Adjusted^b^	1.12 (0.089)	1.06 (0.425)	1.03 (0.701)

*OR* odds ratio

* significantly different from reference group (p < 0.05)

^a^ <3 months recall is the reference group

^b^ Odds ratios were adjusted for: year of death, season (wet/dry), age, education, nationality and place of death. Only in the neonatal group, age was excluded from the model

## Discussion

Although the question as to what might constitute appropriate recall periods for VA interviews has been asked, there is little evidence available on the impact of recall on cause of death outcomes. These analyses from a large dataset covering a long period of time with a wide range of actual VA recall periods go some way to addressing this issue. However, proving that different recall periods have superior performance is difficult with statistical tools; at most and in the absence of a reference standard, one can show that recall periods have equal effects on the VA assessment. Results show very minimal recall effects associated with VAs undertaken anytime during the first year after death, and only small effects during the second year.

Published reports suggest that longer recall periods do not influence reporting of a death event and can be as reliable as short intervals [21, 22]. Using broader categories of the complete WHO 2012 list of VA causes of death, this study attempted to more closely examine the effects of VA recall periods in order to inform best operational practices in HDSSs and mortality surveys.

Minimal recall effects were consistently seen across all cause categories, with the exception of neonatal causes. With most research focusing on recall ability in relation to specific causes such as neonatal deaths [23, 24], operational elements within HDSSs are currently scarce and seen relevant for assessing the overall conclusions on causes of death. The basis for the difference among neonatal causes is not easy to interpret, but may be associated with reluctance to disclose neonatal deaths. Neonatal deaths missed in annual update rounds may therefore be more likely to be discovered in subsequent rounds, consequently have a longer recall period, and therefore contribute to the finding that neonatal causes are associated with longer recall.

Although some categories of causes showed significantly different odds ratios when recall exceeded one year (Table 2), these differences were not seen in the multivariable model, other than for neonatal deaths. It is likely therefore that these recall effects were not independently associated with those cause categories.

Mortality associated with HIV and TB is the leading cause group in this population, and its assessment has been reported to be highly influenced by cultural, environmental as well as operational aspects in other settings [25–27]. Although AIDS-associated stigma has seen a substantial decline in recent years, fear might negatively affect VA outcomes, though our findings do not indicate any specific recall-linked effects.

Seasonal variations in the various underlying aetiologies of respiratory and diarrhoeal infections are very plausible, highlighting the importance of multivariate analysis to adjust for potential confounding in order to correctly interpret these data. To evade associated limitations, a prospectively designed study that randomised identified deaths to a range of VA recall periods could have been implemented in principle, however, this would have been complex and costly, and probably not justifiable. Such a study would certainly not have been done over a twenty year period covering over 10,000 deaths, and we believe that the pragmatic advantages of using this large and rich existing dataset outweigh the theoretical advantages of a more elegantly designed trial.

## Conclusion

VA is becoming an increasingly widely used approach to document uncertified deaths [8, 14] and this study contributes important information about recall effects. With exception for the neonatal causes, our findings clearly show that a recall period of up to one year between death and a VA interview do not have any consequential effects on the cause of death patterns derived. This is an important operational consideration for the widespread used of the VA approach in civil registration, HDSS sites and occasional surveys.
